# “Intrinsic” Anion Exchange Polymers through the Dissociation of Strong Basic Groups: PPO with Grafted Bicyclic Guanidines

**DOI:** 10.3390/membranes9050057

**Published:** 2019-04-29

**Authors:** Riccardo Narducci, Gianfranco Ercolani, Raul Andres Becerra-Arciniegas, Luca Pasquini, Philippe Knauth, Maria Luisa Di Vona

**Affiliations:** 1 Dep. Industrial Engineering, and International Associated Laboratory: Ionomer Materials for Energy, University of Rome Tor Vergata, 00133 Roma, Italy; riccardo.narducci@uniroma2.it (R.N.); raul-andres.becerra-arciniegas@univ-amu.fr (R.A.B.-A.);; 2 Dep. Chemical Sciences and Technologies, University of Rome Tor Vergata, 00133 Roma, Italy; ercolani@uniroma2.it; 3 Aix Marseille Univ, CNRS, MADIREL (UMR 7246) and International Associated Laboratory: Ionomer Materials for Energy, Campus St Jérôme, 13013 Marseille, France; luca.pasquini@univ-amu.fr

**Keywords:** ionomers, ionic conductivity, swelling, acid–base equilibria

## Abstract

We synthesized anion exchange polymers by a reaction of chloromethylated poly(2,6-dimethyl-1,4-phenylene)oxide (PPO) with strongly basic 1,5,7-triazabicyclo[4.4.0]dec-5-ene (TBD). TBD contains secondary and tertiary amine groups in the guanidine portion. To favor the functionalization with the secondary amine, TBD was activated with butyl lithium. The yield of amine formation via the reaction of the benzyl chloride moiety with TBD was 85%. Furthermore, we prepared polymers with quaternary ammonium groups by the reaction of PPO-TBD with CH_3_I. The synthesis pathways and ionomer structure were investigated by NMR spectroscopy. The thermal decomposition of both ionomers, studied by thermogravimetry, started above 200 °C, corresponding to the loss of the basic group. The ion exchange capacities, water uptake and volumetric swelling are also reported. The “intrinsic” anion conductivity of PPO-TBD due to the dissociation of grafted TBD was in the order of 1 mS/cm (Cl form). The quaternized ionomer (PPO-TBD-Me) showed an even larger ionic conductivity, above 10 mS/cm at 80 °C in fully humidified conditions.

## 1. Introduction

The development of anion exchange membranes (AEMs) for electrochemical devices, such as fuel cells, represents an important challenge for many scientists [1,2,3,4,5,6]. The main hindrances for their widespread application are low stability in alkaline conditions, poor hydrophilic/hydrophobic domain separation, the high water content needed to favor the dissociation of the ion pairs, and carbonatation issues that decrease the conductivity, just to mention a few [7,8,9,10,11,12]. These challenges seem very difficult to address, but the benefits of using AEMs justify the efforts. 

AEMs are generally formed by ammonium groups grafted on a polymeric backbone. Hydroxide counter anions are responsible of the conduction and, at the same time, for the degradation of these solid-state ionic materials. The problem is complex and to simplify we can distinguish between the degradation of the polymeric matrix and the degradation of the positive ammonium groups. The most commonly used backbone is poly(2,6-dimethyl-1,4-phenylene)oxide (PPO), a commercial high-performance amorphous polymer that is easy to functionalize in order to form ionomers with a high ion-exchange capacity (IEC). Given that the IEC is inversely related to the equivalent weight (EW) of the polymer repeat unit, a high IEC can be achieved with a low degree of amination, because the EW of the PPO is small. 

PPO is stable under basic conditions, however, the functionalization can alter the charge distribution, making the ether linkages susceptible to the hydroxide attack [13,14,15,16]. Many ammonium groups have been studied, including aromatic and aliphatic molecules linked to the matrix via short or long chains [17,18,19,20,21]. The S_N_2 reaction between the positive nitrogen and the OH^−^ is mainly responsible for the degradation, and it is difficult to prevent [15,22,23,24,25]. In a recent paper, we showed that the stability of the quaternary group can be improved using a bicyclic amine (1,5-diazabicyclo[4.3.0]non-5-ene, DBN) where the delocalization of the positive charge is possible on an extended portion of the molecule [26]. However, the yield of the functionalization reaction is low, because of the high steric hindrance and the scarce nucleophilicity of DBN. 

In this study, we started with chloromethylated PPO and used 1,5,7-triazabicyclo[4.4.0]dec-5-ene (usually abbreviated as TBD, IUPAC abbreviation HPP [27]) as functionalizing agent, which contains secondary and tertiary amine groups in the molecule. TBD is a strong bicyclic guanidine base (pKa = 15.2) protonated in water [28], and soluble in organic media, with a high steric hindrance and the ability, when protonated, to delocalize the positive charge between nitrogens and the double bond. Guanidine bases can act as nucleophiles [27,29], however, to improve the yield of the amination reaction on the secondary nitrogen of chloromethylated PPO, TBD was activated through the reaction with butyl lithium (BuLi). The formed PPO-TBD can behave in water as an “intrinsic” anion exchange membrane due to the strong basic character of TBD. This concept is explored in this work and the properties of PPO-TBD are compared with those of the more classical quaternized PPO-TBD-Me obtained by the methylation of PPO-TBD. 

## 2. Experimental

Poly(2,6-dimethyl-1,4-phenylene)oxide (PPO), 1,5,7-triazabicyclo[4.4.0]dec-5-ene (TBD) and all reagents were purchased from Sigma-Aldrich and used without further purification.

### 2.1. Synthesis of Chloromethylated PPO (PPO-CH_2_Cl)

The procedure was adapted from references [30,31]. The degree of chloromethylation (DCM), determined by NMR spectroscopy, was 0.28.

### 2.2. Synthesis of PPO-TBD Membranes 

TBD (1 g, 7.5 meq) was dissolved in 10 mL of anhydrous THF; then 3 mL of a 2.5 M solution of BuLi in hexane (7.5 meq) was added under N_2_ at 0 °C and stirred for 10 min. A solution of 1 g of PPO-CH_2_Cl (DCM = 0.28, 7.5 meq) dissolved in 10 mL of anhydrous THF at room temperature under N_2_ was prepared separately and added to the BuLi-TBD solutions drop by drop. The resulting yellow solution was heated under stirring at 70 °C for 24 h. After cooling to RT, half of the solution was evaporated under vacuum and the formed solid dissolved in 20 mL of N-methyl-2-pyrrolidone (NMP). The solution was evaporated in a Petri dish at 70 °C and then put in the oven for 48 h at 90 °C. The membrane was peeled off and washed several times in water at 60 °C to remove the excess TBD.

### 2.3. Synthesis of PPO-TBD-Me Membranes 

CH_3_I (1.15 mL, 18.75 meq) was added to the half solution obtained in the previous step (3.4 meq of PPO). The solution was maintained under stirring at RT for 18 h in N_2_ flux. After this time, the resulting solution was dried in the oven at 60 °C for 5 h and the solid dissolved in 20 mL of NMP. The NMP solution was evaporated in a Petri dish at 70 °C and then put in the oven for 48 h at 90 °C. The membrane was peeled off and washed several times in water.

### 2.4. NMR Spectroscopy 

^1^H NMR spectra were recorded with a Bruker AVANCE III spectrometer operating at 400 MHz. DMSO-d_6_ and CDCl_3_ were used as solvent. Chemical shifts (ppm) were referenced to tetramethylsilane (TMS).

### 2.5. Thermogravimetric Analysis (TGA)

High-resolution TGA analysis was performed with a TA Q500 apparatus (TA Instruments, New Castle, DE, USA). The samples were placed in Pt sample holders. The experiments were made under air flux between 50 and 600 °C. The maximum heating rate was 3 K/min.

### 2.6. Ion Exchange Capacity (IEC)

The IEC (in milliequivalents per g of dry polymer) was determined by potentiometric acid-base titration and by Mohr titration. 

*Potentiometric Acid-base Titration.* Membranes were washed in deionized water at 60 °C for 7 days to remove the salts deriving from the synthetic procedure. For the PPO-TBD-Me samples, membranes were immersed 24 h in 2 M KOH at RT and washed in deionized water for 48 h. After drying over P_2_O_5_ for 72 h, the PPO-TBD and PPO-TBD-Me membranes were weighted and immersed in a 0.018 N HCl solution. The acidic solution was then back-titrated with 0.022 N NaOH. 

*Mohr Titration.* For PPO-TBD and PPO-TBD-Me, the chloride forms were obtained by treating the membranes in a 1 M HCl or a 1 M NaCl solution during 48 h, respectively. The membranes were then washed carefully with bi-distilled water to remove the excess Cl^−^. The Cl^−^ anions contained in the membranes were exchanged with SO_4_^2−^ by immersion during 2 days in 1 M Na_2_SO_4_ solution. This solution was then titrated using 0.01 M AgNO_3_ and potassium dichromate as an indicator. The indicator was prepared by dissolving 1 g of K_2_Cr_2_O_7_ in 20 mL of water.

### 2.7. Water Uptake 

The water uptake (WU) was determined at 25 and 60 °C following the equation:

(1) $$\mathrm{WU}=100\times \frac{m_{\mathrm{wet}}-m_{\mathrm{dry}}}{m_{\mathrm{dry}}}$$

m_dry_ is the sample mass after drying over P_2_O_5_ for 96 h. m_wet_ is the sample mass after immersion in deionized water for 48 h. For the measurements at 60 °C, the samples were introduced in closed Teflon vessels and put in a furnace at 60 °C. Before weighing, the excess water at the sample surface was carefully removed with adsorbent paper. 

### 2.8. Volumetric Swelling

The a, b and c dimensions of the samples before and after the immersion (wet) in water were accurately determined at RT with a micrometer. The volumetric swelling was defined as:

(2) $$\Delta V=\frac{a_{\mathrm{wet}}\cdot b_{\mathrm{wet}}\cdot c_{\mathrm{wet}}}{a\cdot b\cdot c}$$

### 2.9. Ionic Conductivity

The membranes in the Cl^−^ form were obtained by treatment in 1 M NaCl solution for 48 h and then washed in deionized water. The fully humidified samples were analyzed by electrochemical impedance spectroscopy (VSP-300, Biologic science instruments, Seyssinet-Pariset, France). This analysis was performed between 1 Hz and 6 MHz with a signal amplitude of 20 mV and stainless steel electrodes inside a hermetically closed Swagelok cell. 

The resistance R of the membranes at 25, 40, 60 and 80 °C was obtained from a Nyquist plot by non-linear least-square fitting using an equivalent circuit composed of a series arrangement of two (resistance-constant phase elements) in parallel [32]. The ionic conductivity was calculated using Equation (3), where d is the membrane thickness and the electrode area A = 0.28 cm^2^:

(3) $$\sigma =\frac{d}{R_{\mathrm{mat}}\cdot A}$$

## 3. Results and Discussion

### 3.1. Synthesis 

**Figure 1:**
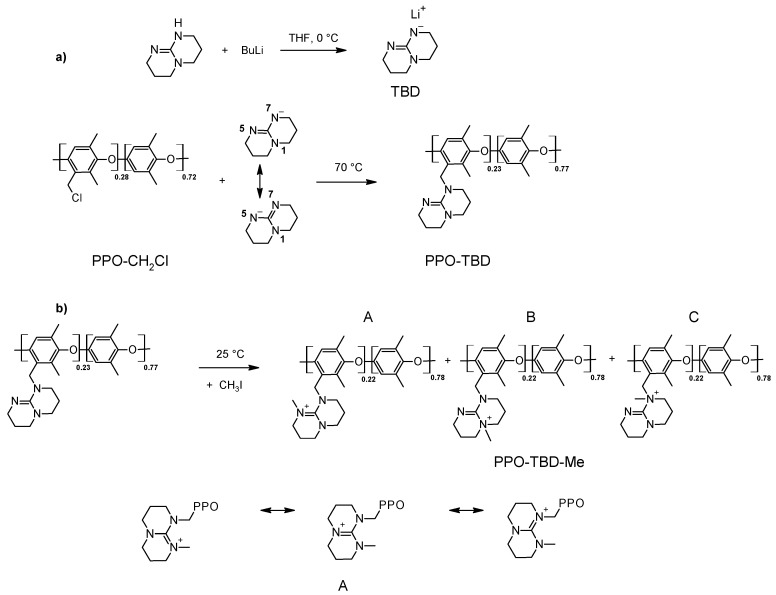
Reaction pathways for the synthesis of (**a**) PPO-TBD and (**b**) PPO-TBD-Me. The different PPO-TBD-Me isomers (A, B, and C) are represented together with the mesomeric formulas of the isomer A.

Figure 1 shows the reaction pathways for the synthesis of TBD-based anion exchange membranes. The amination route was a S_N_2 reaction very sensitive to the steric hindrance and to the strength of the nucleophile. To enhance its nucleophilic properties and to drive the reaction towards the formation of the tertiary amine, TBD was activated with BuLi. The negative charge in the TBD anion was able to be stabilized over the two nitrogens in the symmetrical positions 5 and 7 (Figure 1a). The alkylation of either of the two nitrogens led to the formation of only one isomer, namely PPO-TBD. 

The methylation reaction can occur on different nitrogens, leading to three different isomers (PPO-TBD-Me A, B, and C). However in only one of these (isomer A) was the stabilization of the positive charge possible (Figure 1b). This position is probably the favored position.

**Figure 2:**
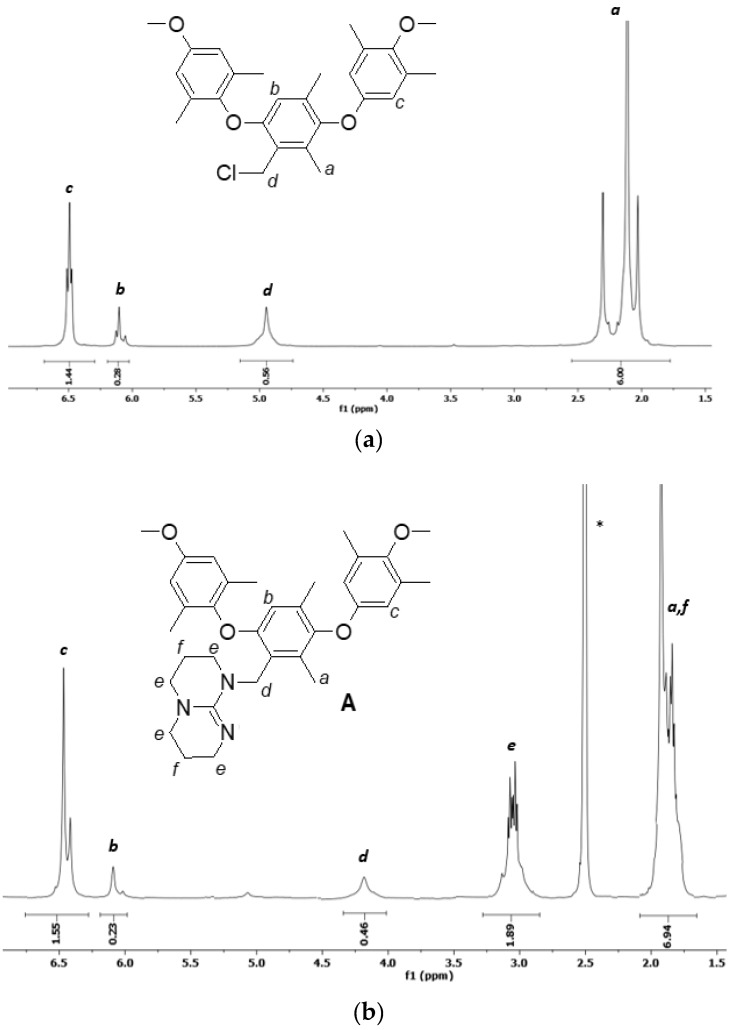

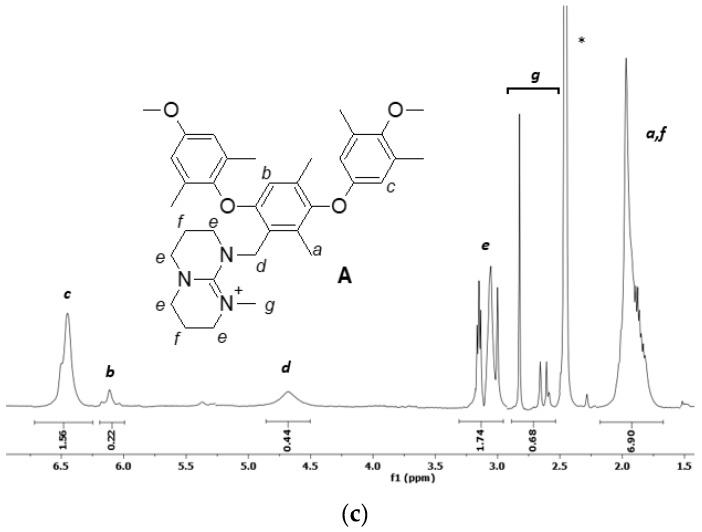
^1^H NMR spectra of (**a**) PPO-CH_2_Cl in CDCl_3_ (the solvent is not shown in the figure, 7.27 ppm), (**b**) PPO-TBD and (**c**) PPO-TBD-Me in DMSO (d_6_). The asterisk indicates the solvent.

The NMR spectra of the precursor and products and the respective assignments are presented in Figure 2.

The spectrum of PPO-CH_2_Cl (Figure 2a) shows the typical signals of benzylic hydrogens (2H) linked to chlorine atom at 4.95 ppm. The integration allowed the calculation of the degree of chloromethylation, taking as a reference the signal of the methyl groups (6H) on the PPO matrix (DCM = 0.28). In the spectra of PPO-TBD and PPO-TBD-Me (Figure 2b,c), the signals *a* (6H) and *f* (4H) overlapped. An estimation of the degree of amination was possible considering that the area of hydrogens *f* was half that of the hydrogens *e* (8H). Subtracting the signal area *f* from the total area of the signal centered around 2.0 ppm (*a* + *f*), it was possible to calculate the area due to hydrogens *a*. For the spectrum 2b, this area corresponded to 5.99 hydrogens, and for the spectrum 2c this corresponded to 6.03 hydrogens in excellent agreement with the expected values. The degree of amination (DA), calculated from the integration of the peak *d* (2H), was equal to 0.23 for PPO-TBD and 0.22 for PPO-TBD-Me. Similar results were obtained by titrations (see below). The spectrum of PPO-TBD-Me (Figure 2c) was more complex due to the coexistence of different isomeric structures, as reported in Figure 1. The signal *g* presented three separate peaks due to the low interconversion between the different isomeric arrangements [33]. This was confirmed by the shift of hydrogens *e*, which experience a different chemical environment. As expected, the integration indicated the presence of only one methyl group on the TBD moiety. The introduction of a further methyl group would lead to the formation of a dication. In the spectra, it was also possible to see a very small peak around 5.4 ppm due to the residual chloromethylated PPO.

### 3.2. Thermogravimetry

**Figure 3:**
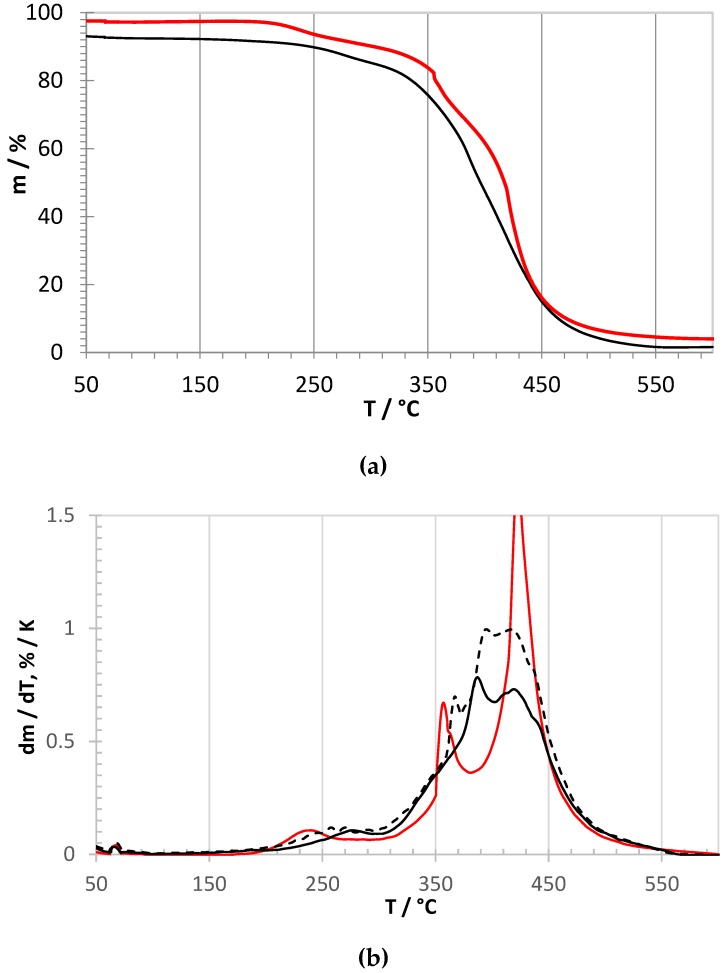
High-resolution thermogravimetric curves under air flow. (**a**) Relative mass loss (black line: PPO-TBD, red line: PPO-TBD-Me). (**b**) Derivative curves. Black lines: PPO-TBD (continuous: pristine, dashed: after 168 h in H_2_O at 60 °C); red line: PPO-TBD-Me).

High resolution thermogravimetric curves for both ionomers are shown in Figure 3. The red curves indicate the quaternized PPO-TBD-Me derivative. One can observe the loss of the TBD group starting above 200 °C, in agreement with data for other amines [34], followed by the loss of the methyl and chloromethyl groups with a maximum at 360 °C [35]. The PPO main chain decomposition had a sharp maximum around 425 °C, in very good agreement with pristine PPO [35,36] and other PPO-based ionomers [14,17]. The black curves correspond to PPO-TBD in pristine form (continuous line) and after 168 h in water at 60 °C (dashed line). The very similar shape of the curves indicates that PPO-TBD supported the prolonged treatment in water without degradation. In comparison with the quaternized ionomer, PPO-TBD presented slightly higher temperatures for the loss of TBD (maximum around 260 °C) and methyl and chloromethyl groups (maximum around 370 °C). The main chain decomposition occurred with two maxima. While the second peak temperature was similar to PPO-TBD-Me (425 °C), the first, with a maximum around 390 °C, can be attributed to in-situ cross-linked macromolecular chains. Similar cross-linking processes by radical species were previously reported in air for other PPO-based polymers [35]. 

### 3.3. Ion Exchange Capacity (IEC)

#### 3.3.1. PPO-TBD

The acid-base titration in 0.018 N HCl, where only the most basic N group was protonated, provided an IEC = 1.3 meq/g. The Mohr titration provided a slightly higher value of 1.6 meq/g, which was however probably overestimated due to the difficult end-point detection. An average IEC = 1.4 meq/g was therefore used for the following calculations. 

The theoretical relation between the IEC and the degree of functionalization DF can be written:

(4) $$\mathrm{IEC}\left(\mathrm{PPO}-\mathrm{TBD}\right)=\frac{\mathrm{DF}}{M\left(\mathrm{PPO}-{\mathrm{CH}}_{2}\right)+\mathrm{DF}\times M\left(\mathrm{TBD}\right)}$$

The theoretical value for one protonated nitrogen is 1.62 meq/g, corresponding to a degree of functionalization of the chloromethylated precursor of 0.28. The experimental value from titration was IEC = 1.4 meq/g, and according to Equation 4 corresponded to a degree of amination of 0.23; The NMR spectroscopy provided an according result (see above). From the comparison of the theoretical and experimental IEC, an amination reaction yield of 85% was deduced, which is quite high for a bulky amine like TBD.

#### 3.3.2. PPO-TBD-Me

The IEC of the quaternized derivative in hydroxide form determined by acid-base titration was (1.20 +/− 0.05) meq/g. The Mohr titration of the chloride form again provided a higher value of 1.5 meq/g. For the same reason as above, an IEC = 1.3 meq/g was used for the calculations. The theoretical IEC corresponding to a degree of functionalization of 0.28 was 1.58 meq/g. The experimental degree of amination of 0.22 (obtained from titration and NMR) was consistent with a yield of 80%, starting from the chloromethylated precursor, slightly below that for PPO-TBD, which is logical, because it also included the quaternization reaction with CH_3_I. 

### 3.4. Water Uptake and Swelling

The water uptake WU and hydration numbers at 20 and 60 °C can be found in Table 1. The hydration numbers λ were calculated using the equation:

(5) $$\lambda =\frac{10\cdot \mathrm{WU}}{\mathrm{IEC}\cdot M\left(H_{2}O\right)}$$

There is no real contradiction between gravimetric water uptake and volumetric swelling data for the two ionomers. The relatively large volumetric swelling of PPO-TBD might be related to a lower cohesion of the polymer network, due to the absence of permanent ionic interactions. The permanent ion charges led to larger hydration numbers for PPO-TBD-Me, but with lower volumetric swelling. The larger hydration number increased the ionic conductivity (see below).

### 3.5. Ionic Conductivity 

**Figure 4:**
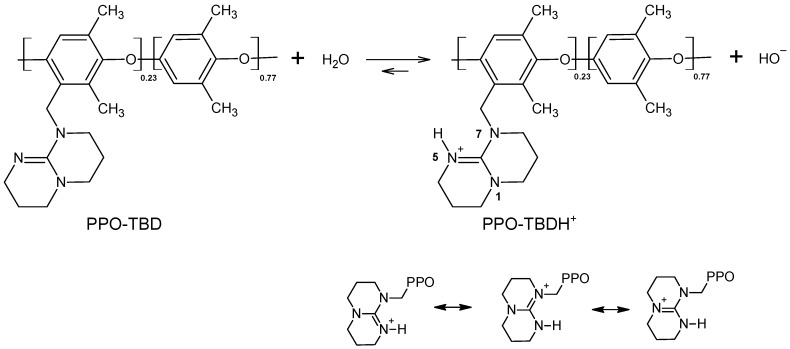
Dissociation acid-base equilibrium of PPO-TBD.

TBD is a very strong base with an experimental acid constant in water pK_a_ = 14.5 [34] (theoretical value: 15.2 [37]). The grafting of the TBD molecule on the PPO macromolecular chain should change the basicity only slightly. The calculated degree of dissociation, according to Figure 4, assuming pK_b_ = −0.5 was 80%. The protonation occurred on nitrogen 5. In this position, the delocalization of the positive charge was possible on the whole guanidine portion (see Figure 4). If the protonation happened on nitrogens in position 1 and 7, TBD would be a much weaker base, like ternary amines. The formed hydroxide anions were able to be exchanged against other anions (such as Cl^−^).

In comparison, the quaternized PPO-TBD-Me samples may present 100% free hydroxide anions, but only if the ion pairs are broken, for example in diluted solution. At high water uptake, the concentration of mobile anions can therefore be up to 20% higher, which should be observable in conductivity. 

Concerning the possible parasitic reactivity of TBD with CO_2_ gas, TBD grafted on a substrate, such as silica [34] or PPO in this work, lost the hydrogen atom of the secondary amine so that carbamate formation is impossible. Carbon dioxide can be bound only by relatively weak Van der Waals interactions, which can be easily overcome. In fact, low CO_2_ absorption rates make tertiary amines difficult to use for CO_2_ gas removal [38]. 

The ionic conductivity of both ionomers in chloride form is reported in Table 2. The interesting concept of an “intrinsic” ionic conductivity by the dissociation of the strongly basic TBD molecule is demonstrated. Given the large difference of ionic mobility between chloride and hydroxide ions, the OH^−^ conductivity is predicted to be above 1 mS/cm at 20 °C. The chloride ion conductivity of the quaternized PPO-TBD-Me is quite high. The difference is larger than expected given the only slightly higher concentration of mobile anions in PPO-TBD-Me. The higher hydration numbers of PPO-TBD-Me are certainly increasing the anion mobility, as previously reported for other proton- and anion-conducting polymers [10,39,40,41]. Furthermore, one can assume a better nanophase separation in quaternized PPO-TBD-Me, due to the permanent ionic charges. The activation energy is very similar for both ionomers, around 0.2 eV.

## 4. Conclusions

The concept of “intrinsic” ionic conducting polymers formed by grafting strongly basic 1,5,7-triazabicyclo[4.4.0]dec-5-ene (TBD) on PPO macromolecules is explored and validated. The ionic conductivity of PPO-TBD is of the order of 1 mS/cm and the membranes are stable during long treatments in water at 60 °C. The more classical quaternized PPO-TBD-Me samples show an even higher ionic conductivity of above 10 mS/cm at 80 °C. These large values are attributed to the larger hydration and better nanophase separation, due to the presence of permanent ionic charges. Thermogravimetric measurements show that both PPO-TBD and PPO-TBD-Me are thermally stable up to 200 °C. 

## Figures and Tables

**Table 1:** Gravimetric water uptake WU, hydration numbers λ and volumetric swelling ΔV after 48 h in H_2_O at 20 and 60 °C of PPO-TBD and PPO-TBD-Me.

Ionomer	T/°C	WU/%	λ	ΔV/%
PPO-TBD	20	19.6	7.8	74
	60	30.0	11.9	85
PPO-TBD-Me	20	32.5	13.9	44
	60	37.5	16.0	47

**Table 2:** Ionic conductivity of PPO-TBD and PPO-TBD-Me (Cl^−^ form) in fully humidified conditions.

T (°C)	PPO-TBD	PPO-TBD-Me
	mS/cm	
20	0.5	4.2
40	0.8	4.8
60	1.0	7.5
80	1.3	11.4
